# Pore‐guided needle insertion: a simple technique to reduce pain during local anesthesia

**DOI:** 10.1111/ddg.16006

**Published:** 2026-02-05

**Authors:** Felipe Bochnia Cerci, Isadora R. Scaburi, Umer Nadir, Stanislav N. Tolkachjov

**Affiliations:** ^1^ Clínica Cepelle Curitiba Brazil; ^2^ Dermatology Service Hospital Universitário Evangélico Mackenzie Curitiba Brazil; ^3^ Epiphany Dermatology, Dallas, Texas; ^4^ Texas A&M College of Medicine, Dallas, Texas; ^5^ Department of Dermatology The University of Texas at Southwestern Medical Center, Dallas, Texas; ^6^ Division of Dermatology Baylor Scott & White, Dallas, Texas

**Keywords:** Dermatologic surgery, local anesthetic, Mohs micrographic surgery, pore‐guided injection, skin cancer

## INTRODUCTION

Local anesthesia is commonly used for a wide range of dermatologic procedures including biopsies, cauterization, excision, and surgical wound reconstruction.^1^ The injection of local anesthetic is often considered the most painful aspect of these procedures and is frequently the event most remembered by patients after the intervention.^2^, ^3^ As such, the reduction of pain associated with local infiltrative anesthesia during dermatologic procedures is essential. Adequate pain control directly contributes to patient satisfaction, reduces perioperative anxiety, and can improve cooperation during the procedure while decreasing the risk of psychological trauma and future aversion to medical care.^4^

The initial insertion of the needle while administering local anesthesia can be painful, especially in areas of the face with high nerve density. While several methods to reduce pain associated with local anesthesia have been described, the authors demonstrate a technique of needle insertion through a pore, which we believe can be an additional method to provide a less painful experience to patients.

## TECHNIQUE

**FIGURE 1:**
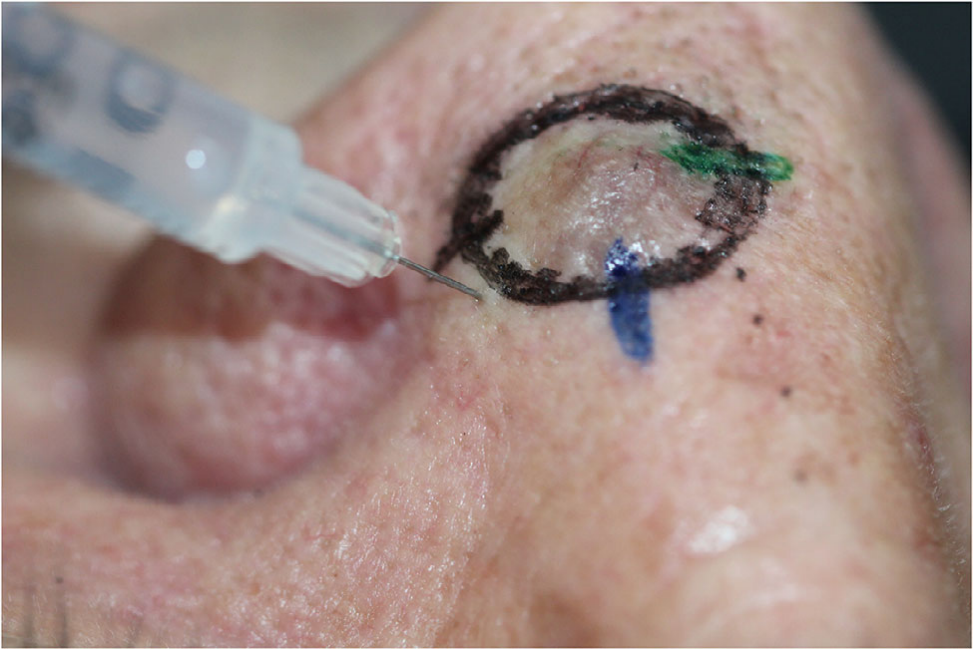
Clinical demonstration of needle insertion through a nasal pore.

After routine antisepsis and identification of an appropriate pore for injection, the needle is injected in a 90‐degree angle through the pore (Figure 1). For better visualization prior to and during injection, dermoscopy, reading glasses (cheaters), or magnifying loupes may be used.

## DISCUSSION

**FIGURE 2:**
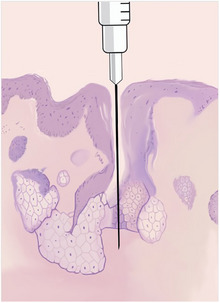
Schematic histology image illustrating the pore insertion “shortcut”.

The authors find this technique useful on sebaceous areas of the face, especially on the distal third of the nose. Inserting the needle for local anesthesia through a pore may result in less pain as it takes advantage of a pre‐existing opening in the skin, thereby causing less disruption to the surrounding tissues. This approach reduces mechanical injury to the dense collagen network and nerve endings found in the dermis, which are primarily responsible for transmitting pain signals (Figure 2). By limiting contact with nociceptors in the superficial dermal layers, the procedure minimizes the initial nociceptive stimulus. Additionally, using the pore as a natural pathway may decrease local inflammation and tissue trauma, contributing to a more comfortable experience for the patient during anesthesia administration. Other techniques to reduce discomfort, such as slow rate of infiltration, using a small gauge needle, using a small syringe, talking to the patient, and distraction techniques, should not be forgotten.^5^, ^6^ Future research should examine the efficacy of this technique and outline further methods for reducing pain with local anesthesia.

## CONFLICT OF INTEREST STATEMENT

S.N.T. is a speaker and investigator for CASTLE Biosciences, Kerecis, and Boehringer Ingelheim. The other authors declare no conflict of interest.
